# Prehospital intravenous fentanyl to patients with hip fracture: an observational cohort study of risk factors for analgesic non-treatment

**DOI:** 10.1186/s13049-017-0348-2

**Published:** 2017-01-19

**Authors:** Kristian D. Friesgaard, Erika F. Christensen, Hans Kirkegaard, Mette D. Bendtsen, Flemming B. Jensen, Lone Nikolajsen

**Affiliations:** 1 Research Department, Prehospital Emergency Medical Service, Central Denmark Region, Olof Palmes Allé 34, 8200 Aarhus N, Denmark; 2 Department of Anesthesiology, Regional Hospital of Horsens, Horsens, Denmark; 3 Department of Clinical Medicine, Pre-hospital and Emergency Research, Aalborg University, Aalborg, Denmark; 4 Department of Anesthesiology and Intensive Care, Emergency Clinic Aalborg University Hospital, Aalborg, Denmark; 5 Unit of Epidemiology and Biostatistics, Aalborg University Hospital, Aalborg, Denmark; 6 Prehospital Emergency Medical Services, North Denmark Region, Aalborg, Denmark; 7 Danish Pain Research Center, Department of Clinical Medicine, Aarhus University, Aarhus, Denmark; 8 Department of Anesthesiology, Aarhus University Hospital, Aarhus, Denmark

**Keywords:** Acute pain, Prehospital, Hip fracture

## Abstract

**Background:**

Patients with proximal femoral neck fracture have a high short-term mortality, a high risk of postoperative complications, and impaired quality of life. One of the challenges related to the prehospital treatment of these patients is to administer systemic opioids fast and properly. Effective analgesic prehospital treatment ought be initiated rapidly in order to alleviate the stress that follows acute pain, to facilitate transportation, and to improve quality of care. The objectives of this study were to explore the prevalence of prehospital administration of intravenous fentanyl to patients with proximal femoral neck fracture in the ambulances and to assess risk factors for analgesic non-treatment.

**Methods:**

This was a register-based observational cohort study of patients with proximal femoral neck fracture from the North Denmark Region transported by ambulance. The patients were identified via the Danish Interdisciplinary Hip Fracture Registry over a 3-year period from 1 July 2011 to 30 June 2014. This hospital registry contains data on several patient characteristics used for the risk factor analysis. Data on prehospital treatment (intravenous fentanyl) and patient monitoring were registered in an electronic prehospital patient record. A modified Poisson regression with robust standard errors was carried out with intravenous fentanyl as the primary binary outcome and the following explanatory variables: age, sex, Charlson Comorbidity Index score, housing, body mass index, type of fracture, fracture displacement, prior consultation with general practitioner, dispatch triage level, and time with ambulance personnel.

**Results:**

In total, 2,140 patients with proximal femoral neck fracture were transported by ambulance, of which 584 (27.3%, 95% CI: 25.4-29.2) were treated with intravenous fentanyl. Risk factors for non-treatment were: older age, male sex (RR 0.77, 95% CI: 0.64-0.91), institutional housing (RR 0.72, 95% CI: 0.56-0.92), medial fracture (RR 0.74, 95% CI: 0.60-0.92), short time with ambulance personnel, Charlson Comorbidity Index score > 1, year of fracture (2011), low levels of urgency at dispatch, and if seen by general practitioners prior to transport.

**Discussion:**

Education of ambulance personnel in assessing and treating patients with hip fracture seems to be required. Also, future studies should consider alternative or supportive pain treatment options with suitable analgesic effects and side effects.

**Conclusions:**

Few patients with proximal femoral neck fracture were treated with intravenous fentanyl, and several risk factors were associated with prehospital analgesic non-treatment. Future prospective studies should explore covariates of socioeconomic, cultural, and psychological origin to provide further insight into the multifactorial causes of non-treatment of acute pain.

## Background

Patients with proximal femoral neck fracture (hip fracture) represent a growing population with high mortality [1], high risk of postoperative complications [2], and impaired quality of life [3]. In acknowledging the challenges related to the treatment of these patients, a list of quality indicators for the treatment of hip fracture has been created to optimize all modifiable factors associated with the quality of care, including the administration of analgesics [4]. Factors associated with early mortality have been extensively explored, including age, sex, comorbidity, type of fracture, time to surgery, housing, and preoperative mobility [5, 6]. Although not directly related to mortality, timely and adequate pain management is essential for early mobilization and recovery [7] and patients with hip fracture will benefit of prehospital treatment started in the prehospital setting.

Only a few older studies have explored acute prehospital pain management in patients with hip fracture, and they report that less than half of patients had been treated with analgesics during ambulance transport [8, 9]. Studies in patients with hip fracture have also been conducted in emergency departments, and the findings consistently report undertreatment of pain in these patients [10–12]. In Denmark, the only available opioid administered by ambulance personnel in the prehospital setting is fentanyl [13, 14]. When dosed and titrated sufficiently, fentanyl is a feasible way of achieving immediate pain relief in patients with hip fracture. However, only a limited number of studies of clinical prehospital care of patients with hip fracture exist. Therefore, the objectives of this study were to determine the prevalence of administration of intravenous fentanyl to patients with hip fracture during ambulance transport in the North Denmark Region and to identify risk factors for analgesic non-treatment. We hypothesized that a low number of patients were treated and that several patient-related factors such as age and comorbidities are associated with non-treatment.

## Methods

### Patients

This is a register-based observational closed cohort study including patients with hip fracture transported by ambulance in the North Denmark Region over a 3-year period from 1 July 2011 to 30 June 2014. The patient cohort was identified via the Danish Interdisciplinary Hip Fracture Registry, which collects national data on quality indicators for the treatment of surgically treated patients ≥ 65 years of age with hip fracure (medial, pertrochanteric, and subtrochanteric). All Danish orthopedic hospital departments caring for these patients are required to report to the registry during patient hospitalization [4]. The registry contains data on patient characteristics, such as the type of fracture and treatment, surgical delay, and body mass index (BMI) [15]. Additionally, the registry has information on the Charlson Comorbidity Index (CCI) score [16] and vital status obtained from the Danish National Patient Registry [17] and the Danish Civil Registration System [18], respectively. This study was approved by the Danish Data Protection Agency (no. 2015-190) and the National Board of Health (no. 3-3013-1424/1). According to the Danish Act on the Scientific Ethical Committee System (law no. 402, paragraph 14, subsection 2), approval of observational studies by the local ethics committee and collection of informed consent are not required.

### Setting

The Danish health care system offers free and unconstrained access to general practitioners (GP), ambulance services, and hospitals to all citizens [19]. The North Denmark Region, which is one of five regions in Denmark, covers a mixed urban and rural area of 8,000 km^2^ and provides healthcare for 588,000 inhabitants. Approximately 800 patients with hip fracture are surgically treated annually at four different hospitals in the region, and most of the patients require ambulance transport from the accident site. The vast majority of patients with hip fracture gain access to acute medical help by dialing the Danish national emergency number (1-1-2) connecting to healthcare professionals in the Emergency Medical Communication Centre (EMCC) who will dispatch an ambulance when needed. Ambulances are dispatched according to a symptom- and criteria-based dispatch protocol which categorizes patients into different levels of urgencies [20]. Alternatively, a GP is contacted and, based on the information obtained from a telephone consultation or a physical examination, the GP can request ambulance transport through the EMCC. All services provided by GPs in the North Denmark Region are prospectively registered with specific codes in a regional electronic database. These registrations are based on a fee-for-service compensation, and the database mainly contains information on the type of consultation (telephone, email, or physical).

Patients with hip fracture transported by ambulance can only be treated with intravenous fentanyl, which is administered by ambulance professionals by delegation of the medical director. All ambulances are staffed with personnel capable of administering fentanyl. Fentanyl is a rapid acting opioid with onset time of 2-3 min and analgesic duration of 7-10 min [21]. Dose adjustments are left to the discretion of the ambulance personnel (maximal dose of 2 μg/kg per transport). It is required to record the treatment effect by assessing the patient’s pain intensity on a numeric rating scale (NRS, 0–10) before initiating fentanyl treatment and every 5 min until hospital admission. Potential fentanyl side effects are monitored by recording vital parameters (respiratory rate, peripheral oxygen saturation, sedation level [Glasgow Coma Scale], blood pressure, and pulse) at frequent intervals. An opioid antidote (naloxone) is administered in the case of an overdose. Data on treatment and patient monitoring are registered in an electronic prehospital patient record (amPHI^®^), whereas technical dispatch data, such as prehospital time stamps and EMCC levels of urgency, are registered automatically in a technical dispatch software (EVA 2000). As all Danish citizens are assigned a personal civil registration number at birth or immigration, a unique individual-level data linkage between these registries is enabled [18].

### Covariates

Data on intravenous fentanyl administration (yes/no), cumulative doses, other medications administered, and vital parameters were collected from amPHI^®^. Prehospital time stamps and EMCC triage levels (A [highest], B, or C) were extracted from EVA 2000. From the Danish Interdisciplinary Hip Fracture Registry, data on age, sex, CCI score (0, 1, 2, or 3+), BMI, type of fracture (medial, pertrochanteric, and subtrochanteric), fracture displacement (yes/no), housing (own home, own home connected with an institution, institution), and relevant dates, such as hospital admission date and operation date, were obtained. Information on whether a GP had examined the patient on the same date as the hospital admission was collected from a regional database. Medicine administered by GPs could be another way for patients to receive opioids and affect the administration of fentanyl in the ambulance.

### Data analysis and handling

All statistical analyses were conducted using STATA version 14.1 (StataCorp, TX, USA). Categorical data are reported as number and percentage (%) with 95% confidence intervals (CIs) and are compared using the *χ* ^2^ test or Fisher’s exact test when appropriate. Continuous outcomes are reported as means with CIs for normally distributed data or as medians with interquartile ranges (IQR) for skewed data and are compared with student’s *t*-test or Mann-Whitney *U*-test when appropriate. A modified Poisson regression with robust standard errors was carried out with intravenous fentanyl as the primary binary outcome and with the following explanatory variables included: age, sex, CCI score, housing, BMI, type of fracture, fracture displacement, prior consultation with a GP, EMCC triage level, and time with ambulance personnel, which was defined as the time from ambulance arrival on the scene until the hospital admission. This model was chosen to obtain interpretable relative risks for frequent events [22]. A standard multivariate logistic regression with odds ratios for the same variables is given in the Appendix. In the adjusted analysis, a likelihood ratio test along with a marginal plot was used to decide that age was included as a continuous explanatory variable and time as a restricted cubic spline due to polynomial development. No imputation for missing data was made. All tests were two-sided and all *P* values < 0.05 were considered statistically significant.

## Results

In the 3-year study period, 2,394 patients from the North Denmark Region aged > 65 years and surgically treated for a hip fracture were identified in the Danish Interdisciplinary Hip Fracture Registry. Of these 2,140 patients (89%) were registered as having been transported by ambulance on the day of their hospital admission. Patients not registered in the electronic prehospital patient record (amPHI^®^) were younger (80.7 years, CI: 79.6-81.7 vs. 83.2 years, CI: 82.9-83.6; *P* = 0.0001), had fewer displaced fractures (73.2% vs. 80.2%; *P* = 0.01), were more likely to have medial fractures (63.4% vs. 50.8%; *P* = 0.003), and were more often examined by a GP on the same day as the hospital admission (31.7% vs. 24.3%; *P* = 0.01). Registered and non-registered patients were similar in terms of 30-day mortality, sex, CCI score, BMI, housing, and reoperation rate due to complications.

Of the 2,140 transported patients with hip fracture, 584 (27.3%, 95% CI: 25.4-29.2) were treated with intravenous fentanyl. The cumulated median dose of fentanyl was 80 μg (IQR 50–100), administered in 2 doses (IQR 1–2). The pain score was documented in 563 of 2,140 patients (26.3%, 95% CI: 24.4-28.2) and mainly if fentanyl was administered (72.6%, 95% CI: 69.0-76.3). Pain scores were more frequently documented for patients with pertrochanteric (27.6%, 95% CI: 24.7-30.6) and subtrochanteric fractures (33.0%, 95% CI: 26.1-39.9) compared with medial fractures (24.1%, 95% CI: 21.6-26.7, *P* = 0.02). For patients with documented pain scores, pain intensity worsened from start of transport (NRS: 5 [IQR 3-8]) to hospital admission (NRS: 6 [IQR 4-8]; *P* = 0.02). No patients were treated with naloxone, and only a few patients received other prehospital treatments that included anticholinergics (*n* = 1), bronchodilators (*n* = 6), antiemetics (*n* = 25), and nitrous oxide (*n* = 16). Risk factors for analgesic non-treatment were older age, male sex (RR 0.77, 95% CI: 0.64-0.91), institutional housing (RR 0.71, 95% CI: 0.56-0.91), medial fracture (RR 0.75, 95% CI: 0.60-0.93), short time with ambulance personnel, low levels of urgency, year of fracture (2011) and if seen by a GP prior to transport. Having at least one comorbidity (CCI score > 1) was associated with analgesic non-treatment (RR 0.83, 95% CI: 0.72-0.96). the same trend was observed when CCI score was divided into 4, although not statistical significant, (Table 1). Estimates stratified by sex, age and comorbidity are given in Table 2. Estimates were not affected by daily, weekly or seasonal variances (data not shown).

**Table 1:** The prevalence of fentanyl treatment and potential risk factors of non-treatment

	*n* = 2140	Fentanyl administered, % (95% CI)	Relative Risk (95% CI)	
			Crude	Adjusted^a^
Age (years), n = 2140				
65-74	362	34.3 (29.3-39.2)	1 (reference)	1 (reference)
75-79	338	30.8 (25.8-35.7)	0.89 (0.72-1.11)	0.90 (0.73-1.12)
80-84	465	26.5 (22.4-30.5)	0.77 (0.63-0.95)	0.77 (0.62-0.95)
85-89	527	24.1 (20.4-27.8)	0.70 (0.57-0.87)	0.72 (0.58-0.89)
> 90	448	23.7 (19.7-27.6)	0.69 (0.55-0.86)	0.81 (0.65-1.00)
Sex, *n* = 2140				
Female	1488	29.0 (26.7-31.3)	1 (reference)	1 (reference)
Male	652	23.3 (20.1-26.6)	0.80 (0.68-0.94)	0.76 (0.64-0.91)
CCI score, *n* = 2140				
0	875	30.9 (27.8-33.9)	1 (reference)	1 (reference)
1	528	23.5 (19.9-27.1)	0.76 (0.63-0.91)	0.77 (0.63-0.93)
2	340	25.0 (20.4-29.6)	0.81 (0.66-0.99)	0.86 (0.69-1.07)
3+	397	26.4 (22.1-30.8)	0.86 (0.71-1.04)	0.90 (0.74-1.09)
Housing, n = 1967				
Own home	1328	30.5 (28.0-33.0)	1 (reference)	1 (reference)
OHATI	210	34.8 (28.3-41.3)	1.14 (0.93-1.40)	1.30 (1.06-1.60)
Institution	429	14.0 (10.7-17.3)	0.46 (0.36-0.59)	0.71 (0.56-0.91)
BMI, *n* = 2140				
Normal	1051	27.0 (24.3-29.7)	1 (reference)	1 (reference)
Underweight	191	26.7 (20.4-33.0)	0.99 (0.77-1.28)	0.99 (0.77-1.29)
Overweight	521	27.8 (24.0-31.7)	1.03 (0.87-1.22)	1.04 (0.88-1.22)
Obese^b^	377	27.6 (23.1-32.1)	1.02 (0.84-1.23)	1.03 (0.82-1.31)
Fracture displacement, *n* = 1981				
Yes	230	23.0 (17.6-28.5)	1 (reference)	1 (reference)
No	1717	27.5 (25.4-29.7)	0.84 (0.65-1.07)	1.07 (0.83-1.36)
Unspecified	34	38.2 (21.0-55.4)	1.39 (0.90-2.14)	1.32 (0.85-2.04)
Type of fracture, *n* = 2140				
Subtrochanteric	182	39.0 (31.9-46.2)	1 (reference)	1 (reference)
Pertrochanteric	872	29.5 (26.4-32.5)	0.76 (0.61-0.93)	0.88 (0.71-1.09)
Medial	1086	23.6 (21.0-26.1)	0.60 (0.49-0.75)	0.75 (0.60-0.93)
Prior consultation with GP, *n* = 2140				
No	1622	30.4 (28.2-32.6)	1 (reference)	1 (reference)
Yes	518	17.6 (14.3-20.9)	0.58 (0.47-0.71)	0.81 (0.66-0.99)
EMCC triage level, *n* = 1895				
A (highest)	145	37.9 (29.9-45.9)	1 (reference)	1 (reference)
B	1246	32.9 (30.3-35.5)	0.87 (0.69-1.08)	1.00 (0.81-1.24)
C	504	11.1 (8.4-13.9)	0.29 (0.21-0.40)	0.43 (0.31-0.60)
Time with ambulance personnel^c^ (minutes), *n* = 1878				
< 20	174	1.7 (0.0-3.7)	0.04 (0.01-0.11)	0.02 (0.00-0.12)
20-29	306	12.4 (8.70-16.1)	0.25 (0.18-0.35)	0.27 (0.19-0.38)
30-39	362	21.0 (16.8-25.2)	0.43 (0.34-0.54)	0.45 (0.36-0.57)
40-49	377	30.0 (25.3-34.6)	0.61 (0.51-0.74)	0.64 (0.53-0.78)
50-59	337	38.0 (32.8-43.2)	0.77 (0.65-0.92)	0.81 (0.68-0.97)
> 60	322	49.1 (43.6-54.6)	1 (reference)	1 (reference)
Year^d^, *n* = 2140				
2011	360	19.7 (15.6-23.9)	1 (reference)	1 (reference)
2012	773	29.5 (26.3-32.7)	1.50 (1.18-1.89)	1.44 (1.11-1.85)
2013	666	28.5 (25.1-32.0)	1.45 (1.14-1.84)	1.41 (1.09-1.83)
2014	341	27.9 (23.1-32.6)	1.41 (1.08-1.85)	1.50 (1.13-2.00)

*CCI* Charlson Comorbidity Index, *OHATI* own home affiliated to an institution, *BMI* Body Mass Index, *GP* general practitioner, *EMCC* emergency medical communication center. The number of complete cases for each variable is given in the left column

^a^Mutually adjusted for the other variables in the table. Adjustments are categorical besides age (continuous) and time with ambulance (restricted cubic splines)

^b^ Four levels of body mass index were defined as < 18.5 (underweight), 18.5-24.9 (normal weight), 25-29.9 (overweight) and > 30.0 (obese)

^c^ Defined as the time from ambulance arrival on scene until hospital admission

^d^ 2011 and 2014 contain patient information from 6-month periods

**Table 2:** Risk factors of fentanyl non-treatment stratified by sex, comorbidity and age

	Male		Female		CCI score 0		CCI score > 1		Age < 85 years		Age > 85 years	
	Fentanyl adm./n (patients)	Adjusted Relative Risk (95% CI)*	Fentanyl adm./n (patients)	Adjusted Relative Risk (95% CI)*	Fentanyl adm./n (patients)	Adjusted Relative Risk (95% CI)*	Fentanyl adm./n (patients)	Adjusted Relative Risk (95% CI)*	Fentanyl adm./n (patients)	Adjusted Relative Risk (95% CI)*	Fentanyl adm./n (patients)	Adjusted Relative Risk (95% CI)*
Housing, *n* = 1967												
Own home	97/391	1 (reference)	309/937	1 (reference)	203/598	1 (reference)	202/730	1 (reference)	260/815	1 (reference)	145/513	1 (reference)
OHATI	22/69	1.36 (0.87-2.13)	51/141	1.28 (1.02-1.60)	29/71	1.41 (1.01-1.95)	44/139	1.27 (0.97-1.66)	35/91	1.26 (0.95-1.69)	38/119	1.26 (0.94-1.71)
Institution	25/136	1.10 (0.73-1.68)	35/293	0.58 (0.43-0.80)	17/140	0.55 (0.34-0.89)	43/289	0.80 (0.59-1.07)	25/163	0.67 (0.46-0.97)	35/266	0.71 (0.51-0.99)
BMI, *n* = 2140												
Normal	69/303	1 (reference)	215/748	1 (reference)	139/454	1 (reference)	34/120	1 (reference)	164/545	1 (reference)	120/506	1 (reference)
Underweight	10/27	1.03 (0.50-2.12)	41/164	0.96 (0.73-1.26)	17/71	0.94 (0.61-1.45)	145/597	1.02 (0.74-1.41)	23/84	0.91 (0.64-1.31)	28/107	1.19 (0.82-1.74)
Overweight	44/198	0.81 (0.56-1.17)	101/323	1.13 (0.94-1.35)	67/206	1.07 (0.85-1.36)	78/315	1.00 (0.79-1.26)	93/308	0.99 (0.80-1.21)	52/213	1.13 (0.86-1.50)
Obese^a^	29/124	1.11 (0.70-1.76)	75/253	0.98 (0.74-1.29)	47/144	1.00 (0.73-.138)	57/233	1.06 (0.76-1.48)	71/228	0.98 (0.75-1.30)	33/149	1.21 (0.78-1.87)
Fracture displacement, *n* = 1981												
Yes	126/519	1 (reference)	347/1198	1 (reference)	221/705	1 (reference)	252/1012	1 (reference)	283/924	1 (reference)	190/793	1 (reference)
No	16/75	0.99 (0.57-1.74)	37/155	1.10 (0.83-1.44)	19/91	0.88 (0.59-1.32)	34/139	1.19 (0.87-1.63)	34/127	1.12 (0.83-1.52)	19/103	0.99 (0.63-1.56)
Unspecified	2/10	0.96 (0.27-3.21)	11/24	1.43 (0.92-2.21)	8/17	1.33 (0.81-2.17)	5/17	1.29 (0.53-3.16)	7/20	1.01 (0.57-1.77)	6/14	1.87 (0.95-3.68)
Type of fracture												
Subtrochanteric	22/60	1 (reference) 0.76	49/122	1 (reference)	33/66	1 (reference)	38/116	1 (reference)	44/102	1 (reference)	27/80	1 (reference)
Pertrochanteric	67/276	(0.49-1.16)	190/596	0.93 (0.73-1.20)	114/353	0.71 (0.52-0.96)	143/519	0.79 (0.58-1.07)	134/444	0.81 (0.61-1.06)	123/428	0.89 (0.62-1.28)
Medial	63/316	0.70 (0.45-1.09)	49/122	0.77 (0.60-0.99)	123/456	0.84 (0.62-1.14)	133/630	0.92 (0.68-1.25)	173/619	0.75 (0.57-0.99)	83/467	0.66 (0.45-0.96)
Prior consultation with GP												
No	116/302	1 (reference)	366/1134	1 (reference)	234/678	1 (reference)	259/944	1 (reference)	303/923	1 (reference)	190/699	1 (reference)
Yes	8/60	0.56 (0.33-0.92)	66/354	0.91 (0.73-1.14)	36/197	0.75 (0.54-1.04)	55/321	0.87 (0.67-1.14)	48/242	0.83 (0.63-1.10)	43/276	0.71 (0.51-0.98)
EMCC triage level												
A (highest)	17/45	1 (reference)	39/88	1 (reference)	32/68	1 (reference)	23/77	1 (reference)	42/106	1 (reference)	13/39	1 (reference)
B	87/202	1.31 (0.84-2.06)	317/897	0.88 (0.70-1.11)	189/516	0.99 (0.76-1.28)	221/730	1.08 (0.75-1.55)	243/679	1.00 (0.77-1.29)	167/567	0.97 (0.66-1.45)
C	10/72	0.68 (0.36-1.27)	37/344	0.34 (0.23-0.50)	19/190	0.32 (0.19-0.55)	37/314	0.55 (0.34-0.88)	32/241	0.47 (0.31-0.70)	24/263	0.40 (0.23-0.72)
Time with ambulance personnel^b^, minutes												
<20	2/29	0.07 (0.01-0.51)	0/125	-	3/67	0.04 (0.01-0.30)	0/107	-	3/88	0.03 (0.01-0.21)	0/86	-
20-29	8/47	0.28 (0.14-0.54)	30/217	0.26 (0.18-0.39)	20/126	0.31 (0.20-0.49)	18/180	0.23 (0.14-0.38)	27/162	0.32 (0.22-0.48)	11/144	0.19 (0.10-0.35)
30-39	15/52	0.62 (0.38-1.00)	55/267	0.42 (0.32-0.55)	39/146	0.53 (0.39-0.73)	37/216	0.38 (0.26-0.54)	44/183	0.46 (0.34-0.62)	32/179	0.45 (0.31-0.65)
40-49	23/57	0.83 (0.56-1.23)	78/255	0.59 (0.47-0.74)	49/161	0.64 (0.48-0.85)	64/216	0.65 (0.50-0.85)	65/196	0.63 (0.49-0.81)	48/181	0.64 (0.47-0.89)
50-59	24/62	0.78 (0.51-1.19)	103/236	0.82 (0.67-0.99)	59/143	0.81 (0.62-1.04)	69/194	0.82 (0.64-1.06)	74/189	0.72 (0.58-0.91)	54/148	0.96 (0.73-1.28)
>60	41/71	1 (reference)	121/218	1 (reference)	68/124	1 (reference)	90/198	1 (reference)	100/198	1 (reference)	58/124	1 (reference)
Year^c^												
2011	20/106	1 (reference)	51/254	1 (reference)	36/163	1 (reference)	35/197	1 (reference)	47/211	1 (reference)	24/149	1 (reference)
2012	61/240	1.44 (0.85-2.44)	167/533	1.47 (1.10-1.96)	106/307	1.60 (1.12-2.31)	122/466	1.24 (0.87-1.78)	145/427	1.43 (1.07-1.92)	83/346	1.47 (0.89-2.43)
2013	51/211	1.23 (0.72-2.11)	139/455	1.50 (1.12-2.01)	81/257	1.49 (1.02-2.17)	109/409	1.30 (0.91-1.87)	109/350	1.33 (0.98-1.81)	81/316	1.61 (0.98-2.64)
2014	20/95	1.48 (0.79-2.76)	75/246	1.55 (1.13-2.13)	47/148	1.60 (1.06-2.41)	48/193	1.37 (0.91-2.04)	50/177	1.28 (0.90-1.81)	45/164	1.96 (1.16-3.31)
Sex												
Female	-	-	-	-	210/666	1 (reference)	222/822	1 (reference)	260/764	1 (reference)	172/724	1 (reference)
Male					60/209	0.83 (0.64-1.07)	92/443	0.71 (0.56-0.90)	91/401	0.70 (0.56-0.87)	61/251	0.89 (0.67-1.19)
CCI score												
0	60/209	1 (reference)	210/666	1 (reference)	-	-	-	-	159/433	1 (reference)	111/442	1 (reference)
1	31/155	0.63 (0.41-0.97)	93/373	0.82 (0.66-1.02)					70/287	0.69 (0.54-0.90)	54/241	0.87 (0.65-1.16)
2	29/121	0.80 (0.54-1.19)	56/219	0.82 (0.63-1.07)					52/204	0.79 (0.61-1.04)	33/136	0.94 (0.65-1.37)
3+	32/167	0.66 (0.44-1.00)	73/230	1.00 (0.81-1.24)					70/204	0.87 (0.69-1.10)	35/156	0.88 (0.63-1.24)
Age, years												
65-74	37/144	1 (reference)	87/218	1 (reference)	67/155	1 (reference)	57/207	1 (reference)	-	-	-	-
75-79	26/108	0.85 (0.53-1.38)	78/230	0.92 (0.72-1.18)	37/115	0.71 (0.50-1.01)	67/223	1.08 (0.80-1.45)				
80-84	28/149	0.64 (0.40-1.04)	95/316	0.84 (0.66-1.06)	55/163	0.78 (0.58-1.05)	68/302	0.78 (0.56-1.08)				
85-89	45/158	0.94 (0.63-1.40)	82/369	0.65 (0.50-0.84)	58/219	0.61 (0.45-0.84)	69/308	0.81 (0.59-1.12)				
>90	16/93	0.59 (0.32-1.11)	90/355	0.86 (0.68-1.09)	53/223	0.76 (0.57-1.01)	53/225	0.90 (0.65-1.28)				

*CCI* Charlson Comorbidity Index, *OHATI* own home affiliated to an institution, *BMI* Body Mass Index, *GP* general practitioner, *EMCC* Emergency Medical Communication Center

*Mutually adjusted for the other variables in the table. Adjustments are categorical besides age (continuous) and time with ambulance (restricted cubic splines)

^a^Four levels of body mass index were defined as < 18.5 (underweight), 18.5-24.9 (normal weight), 25-29.9 (overweight) and > 30.0 (obese)

^b^Defined as the time from ambulance arrival on scene until hospital admission

^c^2011 and 2014 contain patient information from 6-month periods

## Discussion

In this large multicenter, observational study involving 2,140 patients with hip fracture, we found that only 27% of the patients were treated with intravenous fentanyl, and pain scores were high, though only reported in a small fraction of patients. Advanced age, male sex, institutional housing, medial fracture, short time with ambulance personnel, low levels of urgency, year of fracture (2011) and if seen by a GP prior to transport were associated with analgesic non-treatment.

Our results agree with the findings reported in the limited existing literature. A small retrospective medical chart review study of 128 Australian patients with hip fracture found that only half of the patients had received prehospital intravenous morphine [8]. A similar prevalence of prehospital analgesic treatment was found in a more recent multicenter study of 646 patients with hip fracture by reviewing the patterns of prehospital and emergency department analgesia [9]. Neither studies were able to provide a valid assessment of risk factors for analgesic non-treatment.

Platts-Mills and colleagues assessed a large heterogeneous group of prehospital patients transported by ambulance and found a negative association between advanced age (≥65 years compared with 18–64 years) and the likelihood of receiving analgesics [23]. Similar findings have been reported in other acute prehospital and emergency department settings [24–26]. For all five studies, a high number of patients were excluded because of missing data, and their results should therefore be interpreted cautiously. Even though not elucidated clearly in our results, a possible explanation for the low prevalence of treatment among older people is that, they were not asked about pain as indicated by the low number of pain scores. As pain scores were not documented systematically a reliable description of pain intensity was therefore not possible. Hwang et al. systematically assessed 158 patients with hip fracture in an emergency department and found that 81% complained of pain, proposing that the vast majority of these patients still needed analgesic treatment [12]. Our data showed that patients with some types of fractures (medial) were less likely to receive fentanyl and thus less likely to have a documented pain score compared with those with other types (subtrochanteric), suggesting that pain levels vary according to the specific fracture type. Even though these fractures vary within age groups, our analyses were adjusted for multiple confounders, including age. Another explanation for non-treatment, among a subgroup of the old patients may be their inability to express pain and analgesic requirements because of cognitive impairments [11]. This seems to be somewhat supported by our results, indicating that patients living in institutions were less likely to receive fentanyl compared with patients living in their own homes. A third explanation could be the complex age-related pathophysiological changes in the peripheral and central nervous system that modulate the response to noxious stimuli, leading to altered pain perception and lower levels of acute pain [27].

We found that male patients were less likely to receive intravenous fentanyl than women, which has not been observed in similar studies [8, 9]. This finding is in contrast to three larger prehospital studies on patients with acute pain, finding that females were less likely to receive opioid treatment for various reasons [23, 26, 28]. The gender difference in pain research has been extensively explored without reaching any firm conclusions. Some findings show that women report pain more frequently and might experience more severe clinical pain [29]. This could explain the findings in our study if ambulance professionals administered fentanyl on request from female patients.

We found that patients given the lowest level of urgency (C) by the EMCC were less likely to receive fentanyl compared with those given the highest level of urgency (A). One possible explanation might be that patients or relatives that express pain more explicitly tend to be given priority by the EMCC and the treating ambulance professionals. In contrast, one study on 1,246 emergency callers found no association between pain severity and the prioritization of the dispatch responses [30].

Non-treatment was associated with increased comorbidity in terms of CCI score > 1, although not significant when divided into 4 strata (CCI score 0,1,2 and 3+). The CCI score was initially intended to predict in-hospital mortality among medical patients [6, 16] but the potential role of the CCI score in predicting analgesic non-treatment in a prehospital setting has never been explored and should, therefore, be interpreted carefully. It can be argued that patients with comorbidities tend to be more prone to take prescription drugs that potentially interact with opioids. Because ambulance professionals are taught to be cautious with these patients, one would expect to find a lower prevalence of analgesic treatment in patients with comorbidities. The absence of such association can partly be a consequence an insufficient sample size; thus, future research is needed to address this issue. Finally, BMI was not associated with non-treatment, even though it would have been expected that obtaining intravenous access could be more difficult with extreme body weights [31, 32].

As for other observational studies, it can only be speculated why some of the presented explanatory variables are associated with analgesic non-treatment. Acute pain is a subjective experience with multiple biological, socioeconomic, and psychological contributing factors that most likely differ within various strata of patient characteristics, of which some may not be measured quantitatively. The decision of whether to treat acute pain also relies on numerous considerations made by the ambulance personnel, including the fear of inducing adverse effects or priority given to other aspects of treatment when time is critical [33, 34].

Taking these possible explanations into account, continued education of ambulance personnel in sufficiently assessing and treating patients with hip fracture is needed. Additionally, future studies should address alternative/supportive pain treatment modalities with adequate analgesic properties and limited side effects that can be handled satisfactorily by ambulance personnel. Other formulas of opioids with faster onset and shorter analgesic duration could theoretically ease titration [21]. An alternative treatment approach in patients with hip fractures is peripheral nerve blockades, which could prolong the desired analgesic effect and optimize the continuum of patient care from the accident until the initiation of surgery [35]. Nerve blockades could be applied in prehospital settings with advanced care, but this would require discussions of economic and clinical priorities [36–39]. The optimal prehospital treatment in terms of efficacy and safety should be based on results from double-blind randomized controlled trials.

In addition to the large sample size, the strengths of our study are that it included patients who represent real-world population-based data from an entire region. The limitations discussed below reflect its register-based observational design. First, selection was present because patients not registered in the electronic prehospital patient record were younger, had fewer displaced fractures, were more likely to have medial fractures, and were more often examined by a GP on the day of their hospital admission. However, probably this reflects the fact that these patients reached the hospital by means other than ambulance. It can therefore be argued that our final patient cohort represents the typical patient with hip fracture transported by ambulance and that selection bias is a minor concern. Supportively, the population-based design and the free access to health care including emergency medical services minimize the risk of selection bias. Second, information bias cannot be ruled out because erroneous coding of analgesic administration in acute settings is a potential risk [24]. Therefore, the true prevalence of fentanyl administration in ambulances might be slightly higher. Probably, as coding errors would not be unevenly distributed within any of our explanatory variables, potential misclassifications would be non-differential and could bias the results towards the null. Third, although our explanatory variables were adjusted for several potential confounders, relevant unmeasured variables would still be able to affect our estimates. Fourth, in contrast to the treatment protocol prescriptions pain scores were not documented systematically and therefore, a thorough description of pain intensity was not achievable. Imputation was not possible since pain scores were mainly available for patients treated with fentanyl and thereby not missing at random [40]. Last, the association between prior consultation with a GP and lower odds of receiving fentanyl in the ambulance might mirror the fact that patients were treated with opioids by the GP and therefore did not need further analgesics. The database containing information on GP consultations did not have detailed information on treatment, so this factor remains unexplored.

## Conclusion

In this prehospital study exploring transported patients with hip fracture, a small fraction of patients were treated with intravenous fentanyl, and risk factors for analgesic non-treatment included advanced age, male sex, comorbidity, institutional housing, medial fracture, short time with ambulance personnel, low level of urgency assessed at the EMCC, year of fracture (2011) and if seen by a GP prior to transport. These findings give insight into the factors associated with analgesic non-treatment and these need to be taken into account in future studies and clinical care. Improvement of treatment involves several aspects of care, such as education of health care providers and clinical audits on treatment strategies. Future prospective studies should attempt to address covariates of socioeconomic, cultural, and psychological origin to provide further insight into the multifactorial causes of analgesic non-treatment.

## Appendix

**Table 3:** The prevalence of fentanyl treatment and potential risk factors of non-treatment (multivariat logistic regression and estimates as odds ratios)

	*n* = 2140	Fentanyl administered, % (95% CI)	Odds Ratio (95% CI)	
			Crude	Adjusted^a^
Age (years), *n* = 2140				
< 74	362	34.3 (29.3-39.2)	1 (reference)	1 (reference)
75-79	338	30.8 (25.8-35.7)	0.85 (0.62-1.17)	0.80 (0.54-1.20)
80-84	465	26.5 (22.4-30.5)	0.69 (0.51-0.93)	0.62 (0.43-0.91)
85-89	527	24.1 (20.4-27.8)	0.61 (0.45-0.82)	0.54 (0.38-0.80)
> 90	448	23.7 (19.7-27.6)	0.59 (0.44-0.81)	0.65 (0.45-0.97)
Sex, *n* = 2140				
Female	1488	29.0 (26.7-31.3)	1 (reference)	1 (reference)
Male	652	23.3 (20.1-26.6)	0.74 (0.60-0.92)	0.64 (0.48-0.84)
CCI score, *n* = 2140				
0	875	30.9 (27.8-33.9)	1 (reference)	1 (reference)
1	528	23.5 (19.9-27.1)	0.69 (0.54-0.88)	0.64 (0.47-0.87)
2	340	25.0 (20.4-29.6)	0.75 (0.56-0.99)	0.76 (0.54-1.09)
3+	397	26.4 (22.1-30.8)	0.81 (0.62-1.05)	0.83 (0.59-1.17)
Housing, *n* = 1967				
Own home	1328	30.5 (28.0-33.0)	1 (reference)	1 (reference)
OHATI	210	34.8 (28.3-41.3)	1.21 (0.89-1.65)	1.60 (1.09-2.34)
Institution	429	14.0 (10.7-17.3)	0.37 (0.27-0.50)	0.61 (0.43-0.88)
BMI, *n* = 2140				
Normal	1051	27.0 (24.3-29.7)	1 (reference)	1 (reference)
Underweight	191	26.7 (20.4-33.0)	0.98 (0.69-1.39)	1.00 (0.65-1.54)
Overweight	521	27.8 (24.0-31.7)	1.04 (0.82-1.31)	1.07 (0.82-1.43)
Obese^b^	377	27.6 (23.1-32.1)	1.03 (0.79-1.34)	1.07 (0.73-1.56)
Fracture displacement, *n* = 1981				
Yes	230	23.0 (17.6-28.5)	1 (reference)	1 (reference)
No	1717	27.5 (25.4-29.7)	0.79 (0.60-1.09)	1.11 (0.74-1.66)
Unspecified	34	38.2 (21.0-55.4)	1.63 (0.81-3.28)	1.67 (0.68-4.08)
Type of fracture, *n* = 2140				
Subtrochanteric	182	39.0 (31.9-46.2)	1 (reference)	1 (reference)
Pertrochanteric	872	29.5 (26.4-32.5)	0.65 (0.47-0.91)	0.77 (0.51-1.19)
Medial	1086	23.6 (21.0-26.1)	0.48 (0.35-0.67)	0.60 (0.40-0.92)
Prior consultation with GP, *n* = 2140				
No	1622	30.4 (28.2-32.6)	1 (reference)	1 (reference)
Yes	518	17.6 (14.3-20.9)	0.49 (0.38-0.63)	0.74 (0.52-1.02)
EMCC triage level, *n* = 1895				
A (highest)	145	37.9 (29.9-45.9)	1 (reference)	1 (reference)
B	1246	32.9 (30.3-35.5)	0.80 (0.56-1.15)	1.00 (0.66-1.52)
C	504	11.1 (8.4-13.9)	0.20 (0.13-31.6)	0.29 (0.18-0.49)
Time with ambulance personnel^c^ (minutes), *n* = 1878				
< 20	174	1.7 (0.0-3.7)	0.02 (0.01-0.06)	0.01 (0.00-0.06)
20-29	306	12.4 (8.70-16.1)	0.15 (0.10-0.22)	0.14 (0.09-0.22)
30-39	362	21.0 (16.8-25.2)	0.28 (0.20-0.39)	0.27 (0.18-0.39)
40-49	377	30.0 (25.3-34.6)	0.44 (0.33-0.61)	0.45 (0.32-0.63)
50-59	337	38.0 (32.8-43.2)	0.64 (0.47-0.87)	0.67 (0.47-0.95)
> 60	322	49.1 (43.6-54.6)	1 (reference)	1 (reference)
Year^d^, n = 2140				
2011	360	19.7 (15.6-23.9)	1 (reference)	1 (reference)
2012	773	29.5 (26.3-32.7)	1.70 (1.26-2.30)	1.81 (1.21-2.70)
2013	666	28.5 (25.1-32.0)	1.62 (1.19-2.21)	1.73 (1.15-2.60)
2014	341	27.9 (23.1-32.6)	1.57 (1.11-2.23)	1.98 (1.25-3.14)

*CCI* Charlson Comorbidity Index, *OHATI* own home affiliated to an institution, *BMI* Body Mass Index, *GP* general practitioner, *EMCC* Emergency Medical Communication Center. The number of complete cases for each variable is given in the left column

^a^Mutually adjusted for the other variables in the table. Adjustments are categorical besides age (continuous) and time with ambulance (restricted cubic splines)

^b^Four levels of body mass index were defined as < 18.5 (underweight), 18.5-24.9 (normal weight), 25-29.9 (overweight) and > 30.0 (obese)

^c^Defined as the time from ambulance arrival on scene until hospital admission

^d^2011 and 2014 contain patient information from 6-month periods

## Abbreviations

95% CI: 95% Confidence interval
BMI: Body mass index
CCI: Charlson comorbidity index
EMCC: Emergency medical coordination centre
GP: General practitioner
